# Glucosylceramide modifies the LPS-induced inflammatory response in macrophages and the orientation of the LPS/TLR4 complex *in silico*

**DOI:** 10.1038/s41598-018-31926-0

**Published:** 2018-09-11

**Authors:** Edouard Mobarak, Liliana Håversen, Moutusi Manna, Mikael Rutberg, Malin Levin, Rosie Perkins, Tomasz Rog, Ilpo Vattulainen, Jan Borén

**Affiliations:** 1 Laboratory of Physics, Tampere University of Technology, Tampere, Finland; 2 Institute of Medicine, Department of Molecular and Clinical Medicine/Wallenberg Laboratory, University of Gothenburg and Sahlgrenska University Hospital, Gothenburg, Sweden; 3 Department of Chemistry, Indian Institute of Science Education and Research Bhopal, Madhya Pradesh, India; 4 Department of Physics, University of Helsinki, Helsinki, Finland; 5 MEMPHYS-Center for Biomembrane Physics, University of Southern Denmark, Odense, Denmark

## Abstract

Toll-like receptor 4 (TLR4) is activated by bacterial lipopolysaccharide (LPS), which drives the production of proinflammatory cytokines. Earlier studies have indicated that cholesterol- and glycosphingolipid-rich subregions of the plasma membrane (lipid domains) are important for TLR4-mediated signaling. We report that inhibition of glucosylceramide (GluCer) synthase, which resulted in decreased concentrations of the glycosphingolipid GluCer in lipid domains, reduced the LPS-induced inflammatory response in both mouse and human macrophages. Atomistic molecular dynamics simulations of the TLR4 dimer complex (with and without LPS in its MD-2 binding pockets) in membranes (in the presence and absence of GluCer) showed that: (1) LPS induced a tilted orientation of TLR4 and increased dimer integrity; (2) GluCer did not affect the integrity of the LPS/TLR4 dimer but reduced the LPS-induced tilt; and (3) GluCer increased electrostatic interactions between the membrane and the TLR4 extracellular domain, which could potentially modulate the tilt. We also showed that GCS inhibition reduced the interaction between TLR4 and the intracellular adaptor protein Mal. We conclude that the GluCer-induced effects on LPS/TLR4 orientation may influence the signaling capabilities of the LPS/TLR4 complex by affecting its interaction with downstream signaling proteins.

## Introduction

Toll-like receptors (TLRs) are expressed on the surface of sentinel cells such as macrophages and dendritic cells. They recognise broad but highly conserved structural molecular patterns expressed on microorganisms or on endogenous molecules, including those released from damaged tissues^1^. To date, 10 TLRs have been characterized in humans and 12 in mice. Activation of TLRs constitutes the first response of the innate immune system, promoting immediate and efficient proinflammatory and antimicrobial responses^2^. Inappropriate activation of TLRs contributes to prolonged infections, autoimmune diseases, atherosclerosis and various types of cancer^3^.

TLR4 is activated by lipopolysaccharide (LPS), a component of the outer membrane of Gram-negative bacteria, which mediates signal transduction leading to the production of proinflammatory cytokines such as tumor necrosis factor α (TNF-α), interleukin-6 (IL-6) and IL-1^4,5^. In common with the other TLRs, TLR4 is a type I transmembrane protein with an extracellular domain containing leucine-rich repeats (that confer a horseshoe shape where the ligand binds) and a cytoplasmic Toll/interleukin receptor (TIR) signaling domain^6,7^. LPS binds first to the TLR4 co-receptor CD14, which transfers the ligand to the complex formed between TLR4 and its accessory protein myeloid differentiation protein 2 (MD-2) to promote dimerization and thus activation of the TLR4/MD-2 complex^6,7^.

Several studies have shown that cholesterol- and glycosphingolipid-rich subregions of the plasma membrane (lipid domains) are important for proinflammatory TLR4 signaling and that LPS promotes accumulation of TLR4 in these lipid domains^7^. In recent years, sphingolipids [including sphingomyelin, ceramides, glucosylceramide (GluCer), lactosylceramide (LacCer) and complex glycosphingolipids] have emerged as key modulators of many cellular signaling functions, including differentiation, proliferation and immune responses^8^. GluCer synthase (GCS) is the rate-limiting enzyme in the conversion of ceramides to GluCer and downstream glycosphingolipids (Supplementary Fig. S1). GluCer and LacCer, which are present at high concentrations in atherosclerotic plaques^9,10^, play a role in plaque inflammation and vascular smooth muscle apoptosis^11^. We have previously shown that ceramides contribute to the saturated fatty acid-induced proinflammatory response in macrophages^12^. However, the role of glycosphingolipids in the inflammatory response is unclear as both pro- and anti-inflammatory activities have been described for these lipids^13–16^. Furthermore, the role of endogenous glycosphingolipids in the LPS/TLR4-induced proinflammatory response of macrophages has not been investigated.

The aim of this study was to elucidate how GCS-induced changes in the sphingolipid composition of plasma membrane lipid domains affect the LPS/TLR4-induced proinflammatory response in macrophages. We also performed atomistic molecular dynamics simulations to investigate how changes in the sphingolipid composition affect the LPS/TLR4 complex.

## Results

### GCS inhibition reduces LPS/TLR4-dependent cytokine production in macrophages

**Figure 1:**
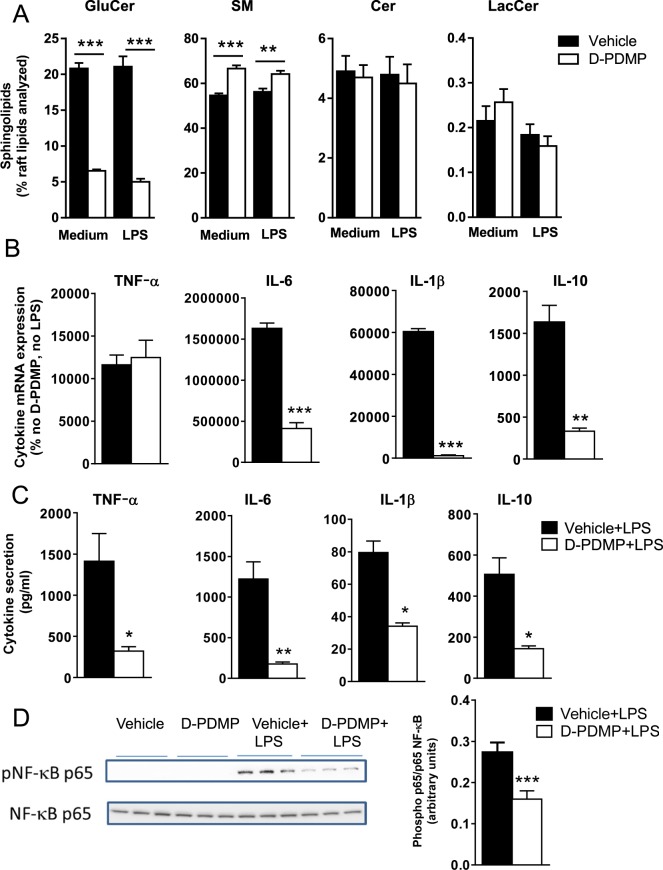
GCS inhibition decreases GluCer concentrations in lipid domains and LPS-induced cytokine production and NF-κB activation in bone marrow-derived mouse macrophages. (**A**) Sphingolipid composition in plasma membrane-isolated lipid domains from macrophages incubated for 24 h in the presence of GCS inhibitor (D-PDMP, 10 µM) or vehicle (ethanol) and thereafter stimulated with 100 ng/ml LPS from *S. typhimurium* and 10 ng/ml INF-γ (or cell medium as control) for 10 min in the continued presence of D-PDMP or vehicle. SM, sphingomyelin; Cer, ceramide. (**B**) Cytokine mRNA expression and (**C**) cytokine secretion from macrophages incubated for 24 h in the presence of D-PDMP (10 µM) or vehicle and thereafter stimulated with 100 ng/ml LPS from *S. typhimurium* and 10 ng/ml INF-γ (or cell medium as control) for 10 h in the continued presence of D-PDMP or vehicle. Results in (**B**) are expressed as % of mRNA expression in control cells (no D-PDMP or LPS). (**D**) Representative immunoblot and quantification showing NF-κB activation in macrophages incubated for 24 h in the presence of D-PDMP (10 µM) or vehicle and thereafter stimulated with 100 ng/ml LPS from *S. typhimurium* and 10 ng/ml INF-γ (or cell medium as control) for 15 min in the continued presence of D-PDMP or vehicle. The images are the crops of the full-length blots presented in Supplementary Fig. S10. Data are mean ± SEM; *p < 0.05, **p < 0.01, ***p < 0.001 versus vehicle, one-way ANOVA followed by Sidak’s multiple comparisons test (A), unpaired t-test (B-D), *n* = 3.

We first investigated the effect of the GCS inhibitor D-threo-1-phenyl-2-decanoylamino-3-morpholino-1-propanol hydrochloride (D-PDMP) on the sphingolipid composition in plasma membrane lipid domains from bone marrow-derived mouse macrophages. As expected, inhibition of GCS with D-PDMP resulted in significantly lower concentrations of GluCer and higher concentrations of sphingomyelin in lipid domains isolated from plasma membrane of macrophages incubated with LPS or cell medium as control (Fig. 1A). Neither ceramide nor LacCer concentrations were affected by D-PDMP in the lipid domains of macrophages incubated either with LPS or cell medium (Fig. 1A). The lack of effect of D-PDMP on LacCer was unexpected given that LacCer is a product of GluCer (Supplementary Fig. S1). However, potential explanations include the fact that endogenous LacCer has a slower turnover rate than GluCer^17^ and/or that LacCer can also be produced by pathways that do not require GluCer as precursor^18^.

Importantly, we observed significant reductions of LPS-induced production of the cytokines IL-6, IL-1β, and IL-10 (both mRNA expression and secretion) and TNF-α (secretion only) in D-PMDP-treated bone marrow-derived mouse macrophages (Fig. 1B,C). LPS/TLR4 is known to promote the expression of proinflammatory cytokines through activation of the transcription factor nuclear factor κB (NF-κB)^19^ and, as expected, we observed that D-PDMP reduced the LPS-induced phosphorylation of NF-κB (Fig. 1D). D-PDMP also significantly reduced LPS-stimulated production of TNF-α, IL-6, IL-1β and IL-10 in human blood-derived macrophages (Supplementary Fig. S2). Of note, flow cytometry showed that D-PDMP did not affect the cell surface TLR4 expression (Supplementary Fig. S3A) nor the binding of LPS-biotin to bone marrow-derived mouse macrophages (Supplementary Fig. S3B).

To confirm the importance of GCS in the LPS/TLR4-induced proinflammatory response, we also knocked down GCS using two different siRNAs in bone marrow-derived mouse macrophages (Supplementary Fig. S4A). This treatment significantly reduced LPS-induced production of IL-6 and IL-1β (both mRNA expression and secretion) and TNF-α (mRNA expression with one siRNA; secretion with both siRNAs) but not IL-10 (Supplementary Fig. S4B,C); the lack of effect on IL-10 is possibly because of incomplete GCS knockdown.

Thus, by using two approaches to reduce endogenous GluCer concentrations in the plasma membrane, we demonstrated the importance of GluCer for the LPS/TLR4-induced proinflammatory response in macrophages.

### TLR4 orientation is affected by LPS and GluCer

**Figure 2:**
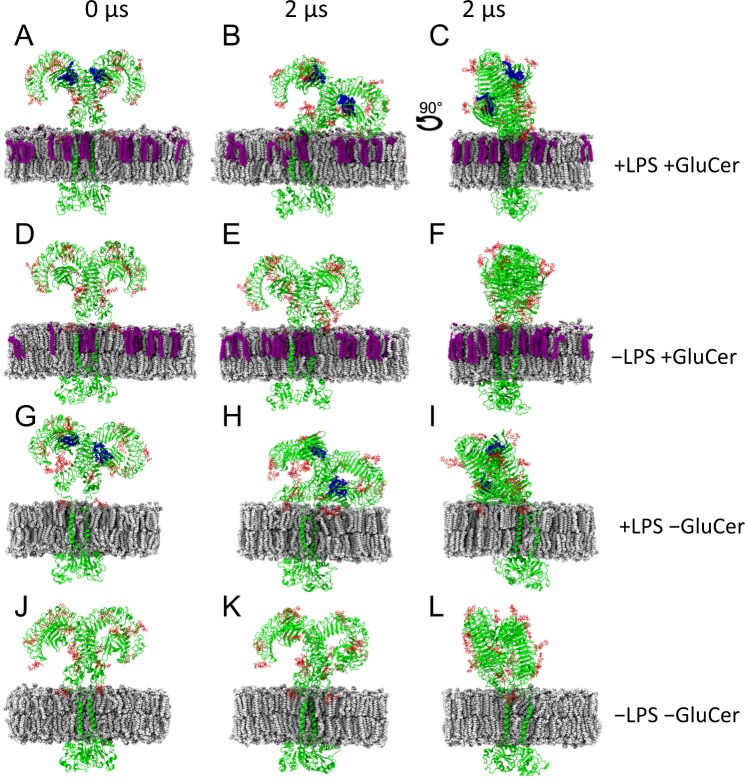
Snapshots from atomistic molecular dynamics simulations show that GluCer affects TLR4 orientation. Snapshots at time 0 (left panels) and at 2 µs (middle and right panels) showing the simulated TLR4 complex in the ‘face’ orientation (when both monomers are aligned to directly face the viewer; left and middle panels) and the ‘side’ orientation (when the ‘face’ is rotated 90°; right panels). (**A–C)** The LPS/TLR4 complex when GluCer is present in the membrane. (**D–F**) The TLR4 complex without LPS and when GluCer is present in the membrane. (**G–I**) The LPS/TLR4 complex when GluCer is absent from the membrane. (**J–L**) The TLR4 complex without LPS and when GluCer is absent from the membrane. Green, TLR4 dimer complex; red, glycans; blue, LPS; white, membrane lipids; purple, GluCer.

To investigate how GluCer affects TLR4, we performed atomistic molecular dynamics simulations of the TLR4 dimer complex (with and without LPS in its MD-2 binding pockets) inserted into a membrane with and without GluCer in its outer fold. ‘Face’ and ‘side’ view snapshots of the TLR4 complex at the end of the 2 µs simulation show that the presence of LPS in the MD-2 binding pocket induced a tilted orientation of TLR4, both when GluCer was present and absent from the membrane (Fig. 2). However, the LPS-induced tilting appears to be less pronounced in the presence of GluCer, particularly when looking at the ‘side’ view (Fig. 2C versus 2I).

**Figure 3:**
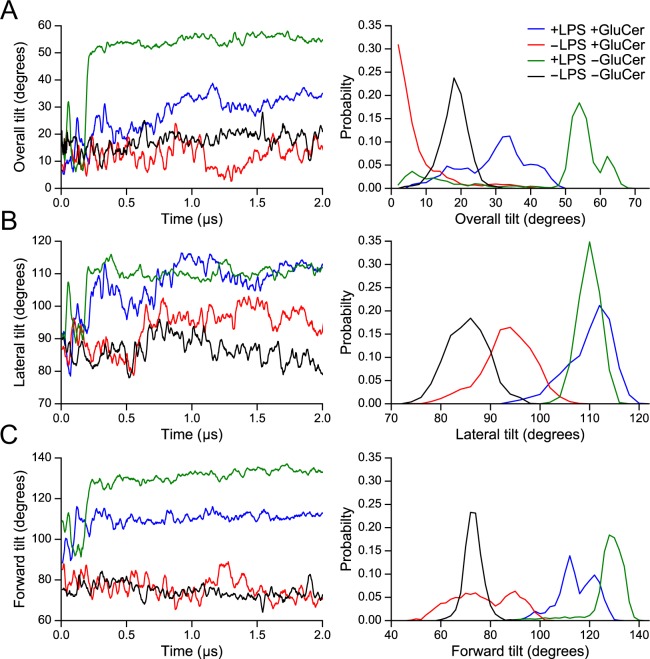
Quantification of tilts from the molecular dynamics simulations of TLR4. Left panels show (**A**) the ‘overall’ tilt, (**B**) the ‘lateral’ tilt, and (**C**) the ‘forward’ tilt of the TLR4 extracellular domain throughout the whole simulation (2 µs) for 1 run in the presence and absence of (1) LPS in the MD-2 binding pocket and (2) GluCer in the membrane. Mean ± EE values of the angles over the last 1 µs of simulation for the 3 runs (1 run for −LPS −GluCer) are presented in Supplementary Fig. S5. Right panels show the normalized angle distribution calculated for the last 1 µs of simulation, averaged over the 3 runs (1 run for −LPS, −GluCer).

To provide a more quantitative description of the different orientations, we defined three vectors (V1-V3; Supplementary Fig. S5A,B) that we used to determine the ‘overall’, ‘lateral’ and ‘forward’ tilts of the extracellular domain of TLR4 (Fig. 3 and Supplementary Fig. S5C). When GluCer was present in the membrane, the ‘overall’ tilt was ~33° and 15° in the presence and absence of LPS, respectively (Fig. 3A). In the absence of GluCer, the ‘overall’ tilt was 57° and 19° in the presence and absence of LPS, respectively (Fig. 3A). The ‘lateral’ tilt of the LPS/TLR4 complex was approximately the same in the presence and absence of GluCer (Fig. 3B), but the ‘forward’ tilt was less pronounced when GluCer was present (Fig. 3C). These results confirm our visual observation (Fig. 2) indicating that LPS induces a more tilted orientation of the TLR4 complex and that the presence of GluCer in the membrane reduces the ‘forward’ tilt of the LPS/TLR4 complex.

For all three tilts shown, the range of angles was greater in the presence of GluCer (Fig. 3). In the absence of GluCer, we mostly observed one sharp peak for the angle distribution, meaning that the tilt of the LPS/TLR4 complex in this case did not vary to any great extent throughout the second half of the simulation.

These differences can be seen more clearly in the Supplementary Videos 1 and 2, which show real-time visualizations of the orientational changes of the LPS/TLR4 complex in the presence and absence of GluCer. We observe that the TLR4 extracellular domain collapses on the membrane and does not tilt back up in the absence of GluCer, whereas in the presence of GluCer, it constantly tilts up and down through the whole simulation. Consistent with the more pronounced tilting in the absence of GluCer, we showed that the number of LPS/TLR4 extracellular domain–membrane contacts was greater when GluCer was absent from the membrane (Supplementary Fig. S6).

**Figure 4:**
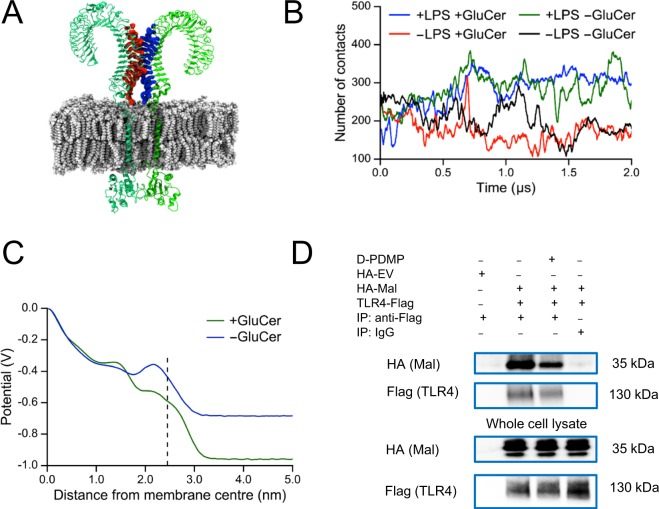
GluCer does not affect dimer integrity but alters electrostatic potential and TLR4 interaction with the intracellular signaling molecule Mal. (**A**) Snapshot of the TLR4 complex showing the regions in the extracellular domain used to calculate the number of contacts between the monomers. A contact was defined as a pair of atoms, each one on a different monomer, within a distance of 0.6 nm. (**B**) Number of contacts between the extracellular domain of the two monomers of TLR4 throughout the whole simulation (2 µs) for 1 run in the presence and absence of (1) LPS in the MD-2 binding pocket and (2) GluCer in the membrane. Mean ± EE values over the last 1 µs of simulation for 3 runs: 337 ± 16 (+LPS +GluCer); 165 ± 14 (−LPS +GluCer); 322 ± 16 (+LPS −GluCer); 187 ± 50 (−LPS −GluCer; 1 run). (**C**) Profile of the electrostatic potential calculated for a symmetric membrane (with the same lipid composition as the outer leaflet of the membrane used in our molecular dynamics simulations of the TLR4 complex) in the presence and absence of GluCer. The dotted line indicates the start of the water phase. The centre of mass of the upright TLR4 extracellular domain is at about 6.5 nm from the membrane centre. (**D**) Representative immunoblots (from two separate experiments) showing co-immunopreciptation of TLR4 with the adaptor Mal. HEK-Blue cells stably transfected with CD14 and MD-2 were treated with 10 µM D-PDMP or ethanol as vehicle for 3 h. Thereafter, the cells were transfected with 8 µg empty vector plasmid HA-EV, or plasmids encoding HA-Mal (4 µg) and TLR4-Flag (4 µg) for 24 h in the continued presence of D-PDMP or vehicle. Thereafter, cells were treated with 100 ng/ml LPS *E. coli* O111:B4 for 10 min. The images are the crops of the expanded blots presented in Supplementary Fig. S11. IP, immunoprecipitation.

To evaluate the integrity of the TLR4 dimer complex in the presence and absence of LPS and GluCer, we calculated the number of contacts between residues of the extracellular domain from each of the two TLR4 monomers (Fig. 4A). The number of contacts increased over time in the presence of LPS and decreased over time in the absence of LPS regardless of whether GluCer was present or absent (Fig. 4B). Thus, the TLR4 complex shows signs of dimer dissociation when LPS is absent, consistent with previous experimental data showing that TLR4 requires LPS to exist as a dimer^6,20,21^. However, it appears that the LPS/TLR4 dimer integrity does not require the presence of GluCer in the bilayer.

We next investigated potential factors that could explain how GluCer affects the orientation of the TLR4 extracellular domain. We speculated that the presence of GluCer could contribute to a reduced tilt by increasing the (repulsive) electrostatic interactions between the overall negatively charged LPS/TLR4 complex and the charged polar lipid headgroups of the membrane. To test this, we calculated the electrostatic potential of a symmetric lipid bilayer (with the same lipid composition as the outer leaflet of our asymmetric bilayer) in the absence and presence of GluCer. In agreement with our hypothesis, we showed that the electronegativity of this lipid bilayer was approximately 30% greater in the GluCer-containing membrane (Fig. 4C). To provide further evidence for a link between GluCer-induced modifications of the electrostatic interactions and the tilt, we carried out additional test simulations by continuing the original four simulations for 200 ns beyond the 2 µs timepoint shown in Fig. 2, this time replacing the particle mesh Ewald (PME) method with the reaction field method to limit the electrostatic interactions. We chose a cutoff of 1.4 nm beyond which electrostatic interactions were neglected. No changes in the protein folding were observed. The ‘overall’, ‘lateral’ and ‘forward’ tilt angles of the extracellular domain of TLR4 are shown in Supplementary Fig. S7. In the presence of LPS (where the TLR4 extracellular domain was already interacting with the membrane at 2 µs), truncating the electrostatic interactions did not affect the angles (i.e., the TLR4 extracellular domain remained tilted) in the presence and absence of GluCer. However, in the absence of LPS (where the TLR4 extracellular domain was upright at 2 µs), truncating the electrostatic interactions increased the tilt angles (i.e., the TLR4 extracellular domain collapsed on the membrane) when GluCer was absent but not when GluCer was present. These results suggest that the short-range repulsive electrostatic interactions between the membrane and the TLR4 extracellular domain are greater in the presence of GluCer, allowing the TLR4 extracellular domain to remain upright when LPS is not present.

We noted that the absence of GluCer affected the conformation of one of the two transmembrane helices of the LPS/TLR4 complex (Supplementary Fig. S8), which could also potentially contribute to the increased tilt of the extracellular domain in the absence of GluCer. To investigate how the presence of GluCer in the bilayer could stabilize the transmembrane helices, we calculated both the bilayer order (of the sphingomyelin acyl chain and backbone) and thickness in the absence and presence of GluCer in a system including the LPS/TLR4 complex. We observed an increase in the bilayer order, and therefore lipid packing, when GluCer was present in the membrane (Supplementary Fig. S9). However, GluCer did not affect the membrane thickness (which was around 4.8 nm in the absence or presence of GluCer) and we did not observe any direct contact between GluCer and the transmembrane helices.

Given that our molecular dynamics simulations showed that GluCer affected both the orientation of the extracellular domain and the conformation of the transmembrane helices of the LPS/TLR4 complex, we speculated that GluCer might also influence the intracellular TLR4 domain and its interaction with downstream intracellular signaling molecules. TLR4 interacts with the adaptor Mal (TIRAP), which in turn recruits the adaptor MyD88 to the TLR4 complex leading to activation of NF-κB and cytokine production^22^. We investigated the effect of D-PDMP on the TLR4-Mal interaction using HEK-Blue cells stably transfected with CD14 and MD-2 and then further transfected with TLR4-Flag and Mal-HA. We showed that coimmunoprecipitation of TLR4 with Mal was reduced in the presence of D-PDMP (Fig. 4D), consistent with our hypothesis that the presence of GluCer in the plasma membrane is important for TLR4 interaction with downstream signaling molecules and thereby affects the signaling capability of the LPS/TLR4 complex.

## Discussion

Here we showed that inhibition of GCS resulted in decreased concentrations of GluCer in plasma membrane lipid domains and reduced the LPS-induced inflammatory response in both mouse and human macrophages. Atomistic molecular dynamics simulations of the TLR4 dimer complex (with and without LPS in the MD-2 binding pockets) in membranes (with and without GluCer in the outer fold) showed that LPS induced a tilted orientation of the extracellular domain of TLR4 (and increased dimer integrity) and that GluCer reduced the LPS-induced tilt. We also showed that GCS inhibition reduced the interaction between TLR4 and the intracellular adaptor protein Mal and therefore propose that the effects of GluCer on LPS/TLR4 orientation influence the signaling capabilities of the LPS/TLR4 complex by affecting its interaction with downstream signaling proteins.

Although accumulating evidence indicates that plasma membranes are laterally heterogeneous, the concept of highly ordered lipid domains that act as a platform for membrane-associated signaling proteins remains a hotly debated topic^23^. Results supporting a role for plasma membrane lipid domains in LPS-induced TLR4 signaling are mainly from studies using Triton X-100 to obtain detergent-resistant membrane fractions, although evidence has also been obtained from studies using fluorescence resonance enery transfer and fluorescence recovery after photobleaching^7^. The validity of the Triton X-100 method has been questioned because this non-ionic detergent may create ordered domains in an otherwise homogenous membrane^24^. Therefore, it is important to note that the detergent-resistant fraction should not be assumed to be identical to plasma membrane lipid domains in unperturbed cells.

By using two approaches (a GCS inhibitor and GCS knockdown) to reduce endogenous GluCer concentrations in the plasma membrane, we showed the importance of GluCer for the LPS/TLR4-induced proinflammatory response in macrophages. Our results contrast with those of earlier studies showing that glycosphingolipids have anti-inflammatory effects. In a mouse macrophage cell line, GluCer decreased the LPS-induced activation of NF-κB, MAP kinase pathways and cytokine production (TNF-α and IL-1β)^13^. Furthermore, in neuronal and epithelial cell lines, complex glycosphingolipids (the gangliosides GM1 and GD1a) reduced the LPS-induced production of inflammatory mediators and translocation of TLR4 to the lipid domains^25^. However, both of these earlier studies used exogenously added glycosphingolipids, which likely have a direct signaling effect on the cells. In addition, the GluCer used in the mouse macrophage study was from plants and thus has a different sphingosine backbone and possible function than GluCer of animal origin^14,26^.

Our molecular dynamics simulations of the TLR4 dimer complex showing that the presence of LPS in the MD-2 binding pockets induced a tilted orientation of the TLR4 extracellular domain are consistent with results from a recently published study that used molecular dynamics simulations to model the LPS/TLR4 dimer complex^27^. Although Patra *et al*. did not perform simulations in the absence of LPS, they proposed that the presence of LPS in the MD-2 binding pockets induces localized changes in the charges of the TLR4 extracellular domain and an increase in electrostatic interactions with the membrane that contribute to a tilted orientation of the receptor. They also suggested that the interaction between the negatively charged phosphate groups of lipid A (the active component of LPS) and the positively charged arginine and lysine clusters present on the TLR4 complex provide support for receptor dimerization, again consistent with our observation of increased dimer integrity in the presence of LPS. However, it is important to note that comparisons between the two studies are limited because the earlier study used a membrane system that was much simpler than ours and it did not include GluCer, and TLR4 was not glycosylated whereas it was in our simulation model.

Our simulations showed that the LPS-induced tilt of the TLR4 extracellular domain was reduced when GluCer was present in the membrane. Given that GluCer is a glycolipid with a small and uncharged headgroup and TLR4 is a large negatively charged protein, direct interactions between GluCer and the TLR4 extracellular domain are unlikely to explain this stabilizing effect of GluCer. In an earlier simulation study, we showed that the composition of lipids in the membrane affected the orientation of the CD2 extracellular domain and that these lipid-induced changes were driven by altered electrostatic interactions without any significant contribution from altered lipid-protein interactions^28^. It is known that glycolipids affect the physicochemical properties of lipid bilayers^29^ and increase the electrostatic potential difference between water and the water-membrane interface^30^, which led us to speculate that GluCer might affect the orientation of TLR4 by altering the electrostatic potential of the membrane. Indeed, we showed that the electrostatic potential of symmetric lipid bilayers was approximately 30% greater in the GluCer-containing membrane. In addition, by neglecting electrostatic interactions beyond a cutoff of 1.4 nm, we showed that the short-range repulsive interactions between the membrane and the TLR4 extracellular domain appeared to be greater in the presence of GluCer. Thus, we propose that the increased (repulsive) electrostatic interactions between the negatively charged LPS/TLR4 complex and the membrane contribute to a less pronounced tilt of the LPS/TLR4 complex in the presence of GluCer.

In our simulations, we also noted that the conformation of one of the two transmembrane helices of the LPS/TLR4 complex was altered when GluCer was absent from the membrane. This response could be a consequence of the increased tilt in the extracellular domain in the absence of GluCer but could also indicate that GluCer has a stabilizing effect on the transmembrane helices through modification of the membrane properties. In agreement with previous findings^31^, we showed that the presence of GluCer in the membrane increased the lipid order and therefore lipid packing, which could contribute to the stability of the helices and thereby to a reduction in the LPS-induced tilt of the TLR4 extracellular domain. Patra *et al*. also observed deformation of the transmembrane helices in their molecular dynamics simulations of the LPS/TLR4 dimer complex, which they proposed was caused by hydrophobic mismatch with the bilayer core^27^. However, as indicated above, the different lipid composition of their membrane makes direct comparisons with our study inappropriate. In contast to the liquid-disordered state of their lipid bilayer, ours was in a liquid-ordered state. Our transmembrane helices were therefore completely buried inside the bilayer thickness, and were more restricted by densely packed lipids. Therefore, we do not consider that hydrophobic mismatch would cause deformation of the transmembrane helices in our study.

In conclusion, we combined experimental and computer simulation studies and showed that a change in membrane lipid composition (i.e., absence versus presence of GluCer) affected both the LPS-induced inflammatory response in macrophages and the orientation of the LPS/TLR4 dimer complex. We also showed that GCS inhibition (and thus reduction of GluCer in the plasma membrane) reduced the interaction between TLR4 and the intracellular adaptor protein Mal. Further studies are required to determine whether the GluCer-induced changes in the orientation and conformation of the LPS/TLR4 complex influence downstream signaling by, for example, affecting the position and orientation of the intracellular TIR domain.

## Methods

### Ethics and adherence to guidelines and regulations

All methods and experiments were carried out in accordance with relevant guidelines and regulations. All animal procedures were approved by the Research Animal Ethics Committee in Gothenburg, and adherence to the principles of the 3Rs: reduction, refinement and replacement.

### Cell culture and stimulation

Hematopoietic stem cells were isolated from bone marrow cells extracted from the femurs and tibias of 10-12-week-old male C57BL/6 mice using the EasySep® Mouse Hematopoietic Progenitor Cell Enrichment Kit (Stem Cell Technology). Purified cells were differentiated to macrophages for 6–7 days in cell medium (high-glucose DMEM supplemented with 10% fetal bovine serum (FBS), 50 µg/ml gentamycin and 50 µmol/l β-mercaptoethanol) containing either recombinant macrophage colony-stimulating factor (M-CSF) 50 ng/ml (R&D Systems) or 10% CMG cell supernatant as source of M-CSF (cell complete medium). Human blood-derived peripheral blood mononuclear cells were obtained from buffy coats of healthy individuals and differentiated to macrophages using granulocyte macrophage colony-stimulating factor as previously described^12^.

Unless otherwise indicated, cells were incubated for 24 h in the presence of 10 µM D-PDMP (Matreya) or ethanol as vehicle in cell complete medium. Thereafter, cells were stimulated with either 100 ng/ml LPS from *Salmonella typhimurium* (Sigma) and 10 ng/ml interferon γ (INF-γ; R&D Systems) or 100 ng/ml LPS from *Escherichia coli* O111:B4 (ultrapure; InvivoGen) as indicated in the figure legend, or cell medium as control, in the continued presence of 10 µM D-PDMP or ethanol.

### Lipid analysis

Plasma membrane was isolated from macrophages as described previously^32^. Lipid domains were obtained from plasma membrane using 1% Triton X-100 as described earlier^33,34^. Lipids were extracted from lipid domains using the Folch procedure^35^ and analyzed as previously described^36^.

### RT-qPCR expression analyses

RNA was isolated from macrophages using RNeasy Mini Kit according to the manufacturer’s instructions (Qiagen). Double-stranded cDNA was generated from RNA using a kit from Applied Biosystems. Real-time PCR amplification and analysis was conducted using the 7900HT Real-Time PCR system (Applied Biosystems). The standard amplification protocol was used with pre-designed probe sets (Applied Biosystems) and TaqMan Fast Universal PCR Master Mix (2X; Applied Biosystems). Cytokine mRNA levels were normalized to 36B4 (for mouse macrophages) or 18S gene expression (for human macrophages) using the ΔCt method.

### Cytokine secretion analysis

Cytokine secretion in cell supernatant was analyzed by ELISA using Proinflammatory Cytokine Mouse Kits from Mesoscale according to the manufacturer’s instructions.

### Transfection with siRNA

Macrophages were plated at a density of 2 × 10^5^ cells per 6 well plate and transfected the following day with 100 pmol/l siRNA that targets the GCS gene (*ugcg*, Ambion; ID s75735 and s75734) or scrambled control siRNA using Lipofectamine™ RNAiMAX (Invitrogen) according to the manufacturer’s protocol. 48 h after transfection, cells were treated with 100 ng/ml LPS (from *S. typhimurium*) and 10 ng/ml recombinant mouse INF-γ.

### Immunoblotting

Macrophages were lysed in lysis buffer (Cell Signaling) containing protease and phosphatase inhibitors (Roche). Protein concentration of cell lysates was measured by using the Pierce BCA protein assay kit (Thermo Fisher Scientific). Equal amounts of protein were loaded on NuPAGE 4–12% Bis-Tris gel (Invitrogen) and transferred thereafter to a nitrocellulose membrane. Membranes were blocked with 5% BSA and probed overnight with antibodies against pNF-κB p65 (Cell Signaling) followed by the corresponding horseradish peroxidase-conjugated secondary antibody. Immunoblots were developed with Immobilion Western Chemiluminescent Horseradish Peroxidase Substrate (Millipore) and visualized with a digital camera.

### Flow cytometry

The cell surface expression of TLR4 was determined by flow cytometry with TLR4-APC conjugated antibodies (clone SA15–21; Biolegend); rat IgG2a-APC conjugated (Biolegend) was used as isotype control. 50,000 events were recorded using an Accuri C_6_ Flow Cytometer.

### LPS binding to macrophages

After incubating macrophages for 24 h in the presence or absence of 10 µM D-PDMP, 1 × 10^6^ cells were incubated in suspension for 15 min in the presence or absence of 50 µg/ml LPS-biotin followed by washing in PBS and incubation with 10 µg/ml streptavidin-Alexa fluor 488 on ice for 15 min as indicated in the protocol provided by InvivoGen. The fluorescent signal was analyzed by flow cytometry using an Accuri C6 Flow Cytometer. The results were expressed as median fluorescence intensity.

### Atomistic molecular dynamics simulations of TLR4

The TLR4 homodimer model was constructed based on the 835 residues sequence of mouse TLR4 (Uniprot id: Q9QUK6)^37^. The structure of the TLR4 extracellular domain in a complex with the adaptor molecule MD-2 and the LPS precursor lipid IVa (in the MD-2 binding pockets) was taken from the crystal structure (PDB id: 3VQ1)^38^. Lipid IVa was converted into lipid A by the addition of two 14-carbon-long acyl chains. The intracellular TIR domain was constructed by homology modelling using other TLRs and molecules featuring a TIR-like domain as templates. Twenty tentative models (see Supplementary Table S1) were created using the SWISS-MODEL modeling platform (https://swissmodel.expasy.org)^39–43^, and the three best models were then used as templates to create a refined model with the Modeller software (https://salilab.org/modeller)^44–48^. The trans-membrane helix was modelled *de novo*. The Modeller software was used to co-assemble the extracellular domain (601 residues), the trans-membrane helix (21 residues), and the TIR domain (176 residues), and to add the missing 11 residues between the extracellular domain and the trans-membrane helix; these 11 residues were modelled as a random loop.

The TLR4 glycosylation sites were identified and glycans were attached using the doGlycans package^49^. Only the core glycans (GlcNAc2Man3) were attached because of a lack of information about the full structure of glycans. The complete list of glycosylated residues is in Supplementary Table S2. A variant of the TLR4 model lacking LPS was also constructed.

Following a procedure described earlier^50^, we constructed two asymmetric lipid membranes: (1) to mimic the cellular membrane with an outer fold composed of 45 mol% cholesterol, 25 mol% sphingomyelin, 10 mol% GluCer and 20 mol% phosphatidylcholine, and an inner fold composed of 35 mol% cholesterol, 20 mol% phosphatidylserine, 20 mol% phosphatidylethanolamine and 25 mol% phosphatidylcholine; and (2) a variant of the first with an outer fold lacking GluCer and instead composed of 45 mol% cholesterol, 35 mol% sphingomyelin and 20 mol% phosphatidylcholine (the inner fold was not modified). TLR4 (with or without LPS) was inserted into the bilayers (with and without GluCer in the outer fold) following a procedure described elsewhere^51^.

The TLR4/membrane systems were hydrated with approximately 150,000 water molecules. Salt ions (NaCl) were added at a physiological concentration of 154 mM, and counter-ions were added to neutralize the system charges. The numbers of each type of molecule in the four systems are listed in Supplementary Table S3. All molecules were parameterized using the OPLS-AA force field^52,53^, with additional parameters specifically derived for lipids^54–57^. The TIP3P model compatible with the OPLS force field^58^ was chosen as the water model. Finally, partial charges for LPS were calculated following the OPLS-AA procedure.

The TLR4/membrane systems +LPS +GluCer, −LPS +GluCer, +LPS –GluCer were simulated in three runs for 2 µs each using the GROMACS MD simulation package version 4.6.7 (www.gromacs.org/About_Gromacs). The system –LPS –GluCer was simulated for 2 µs only once because the simulation showed that TLR4 in this system was exceptionally rigid and remained in an upright position, with no strong driving force for conformational fluctuations in the extracellular domain (Fig. 2J–L). The LINCS algorithm^59^ was used to preserve the length of covalent bonds between heavy atoms and hydrogen atoms. The simulation program reported in this paper took about 3000 core-years of computing resources. Simulations were performed with a timestep of 2.0 fs, under a constant temperature of 310.0 K maintained via the V-Rescale thermostat^60^, and a constant pressure of 1.0 atm maintained via the Parrinello-Rahman barostat^61^, with time constants of 0.1 ps and 2.0 ps, respectively. A semi-isotropic scheme was used for pressure control, and the temperatures of water, membrane, and protein complex were controlled separately. A 1.0 nm cut-off was used for the Lennard-Jones interactions, and electrostatic interactions were evaluated with the PME algorithm^62^ with real space cut-off of 1 nm. Finally, the list of nonbonding interactions was updated every 10 steps.

We also continued the original four simulations (for the TLR4/membrane systems +LPS +GluCer, −LPS +GluCer, +LPS −GluCer and −LPS −GluCer) in one run for 200 ns beyond the 2 µs timepoint using the reaction field method^63^, with a cutoff of 1.4 nm instead of the PME algorithm to evaluate the electrostatic interactions. All other simulation parameters remained the same.

To evaluate the effects of GluCer on the electrostatic potential of the lipid bilayer, we created two symmetric membranes (without TLR4) using the lipid compositions given for the outer fold in the TLR4 simulations above. The membranes were hydrated with about 13,000 water molecules, and salt ions (NaCl) were added at a physiological concentration of 154 mM. Both membranes were then equilibrated for 100 ns. We calculated the electrostatic potential running through both membranes using the g_pot GROMACS tool by computing the charge density of the whole simulation box, and then integrating it twice over the Z coordinate across the membrane.

### Coimmunoprecipitation in HEK-Blue cells

HEK-Blue cells stably transfected with human CD14 and MD-2 (InvivoGen) were plated in 10 cm dishes (3.5 × 10^6^ cells/dish). After 24 h, cells were treated with 10 µM D-PDMP or ethanol as vehicle for 3 h. Cells were transfected thereafter with 8 µg empty vector plasmid or plasmids encoding 4 µg HA-Mal (both kindly provided by Luke O´Neill, Trinity College Dublin, Ireland) and 4 µg TLR4-Flag (Addgene) for 24 h in the continued presence of D-PDMP or vehicle. 24 h after transfection, the cells were treated with 100 ng/ml LPS from *E. coli* O111:B4 for 10 min and lyzed with 700 µl of high stringency buffer (50 mM Tris-HCl pH 7.5, 150 mM NaCl, 2 mM EDTA and 1% Igepal). After removing the cell debris by centrifugation (10,000 *g*, 10 min) the cell lyates were adjusted to the same protein concentration. 50 µl cell lysate was saved as whole cell lysate. The remaining lysate was immunoprecipitated with anti-Flag antibodies (clone M2 Sigma) or mouse IgG1kappa isotype control (Biolegend). Western blot was performed with antibodies against HA (Biolegend) to detect Mal and Flag to detect TLR4.

### Statistical analysis

Experimental data are presented as mean ± SEM. Statistical analyses were performed using GraphPad Prism. Comparisons between two groups were done using unpaired two-tailed t-test. Comparisons between more than two groups were analyzed by using one-way analysis of variance (ANOVA) followed by Sidak’s or Dunnett’s post-hoc comparison tests. A p value of <0.05 was considered statistically significant. Data from the molecular dynamics simulations are presented as mean ± error estimations (EE) calculated using the block averaging method as described^64^.

## Electronic supplementary material

Video 1

Video 2

Supplementary file

## Data Availability

The datasets generated during and/or analyzed during the current study are available from the corresponding author on reasonable request.
