# Supplementary material for: Glucosylceramide modifies the LPS-induced inflammatory response in macrophages and the orientation of the LPS/TLR4 complex in silico
Source: Sci Rep. 2018 Sep 11;8:13600. doi: 10.1038/s41598-018-31926-0 (PMC6134110; doi:10.1038/s41598-018-31926-0)
Supplement: Supplementary file 3 — Supplementary file [file 41598_2018_31926_MOESM3_ESM.pdf]

Supplementary Information

Glucosylceramide modifies the LPS-induced inflammatory response in macrophages and the orientation of the LPS/TLR4 complex *in silico*

Edouard Mobarak[1,6], Liliana Håversen[2,6], Moutusi Manna[1,3], Mikael Rutberg[2], Malin Levin[2], Rosie Perkins[2], Tomasz Rog[1,4,7], Ilpo Vattulainen[1,4,5,7,*], and Jan Borén[2,7,8,*]

[1]Laboratory of Physics, Tampere University of Technology, Tampere, Finland
[2]Department of Molecular and Clinical Medicine/Wallenberg Laboratory, University of Gothenburg and Sahlgrenska University Hospital, Gothenburg, Sweden
[3]Department of Chemistry, Indian Institute of Science Education and Research Bhopal, Madhya Pradesh, India
[4]Department of Physics, University of Helsinki, Helsinki, Finland
[5]MEMPHYS-Center for Biomembrane Physics
[6]These authors contributed equally to this work
[7]Senior authors
[8]Lead contact

***Correspondence:**

Jan Borén, MD, PhD
Wallenberg Laboratory
Sahlgrenska University Hospital
413 45 Gothenburg, SWEDEN
Tel: +46 733764264
jan.boren@wlab.gu.se

Ilpo Vattulainen
Department of Physics
POB 64, FI-00014 University of Helsinki
FINLAND
Tel: +358 400 510 592
ilpo.vattulainen@helsinki.fi

Supplementary Figure S1

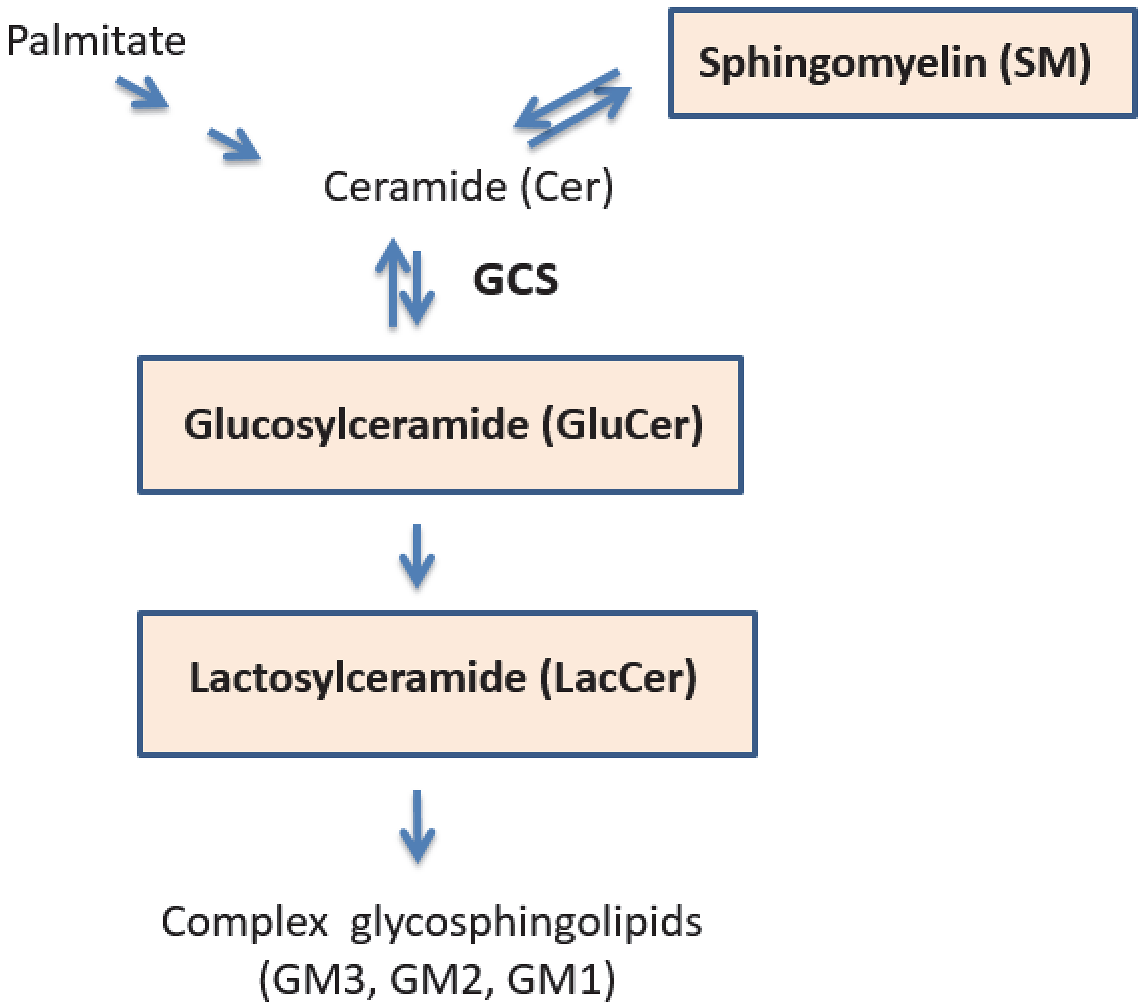

Supplementary Figure S1. Schematic illustration of the sphingolipid pathway.

Supplementary Figure S2

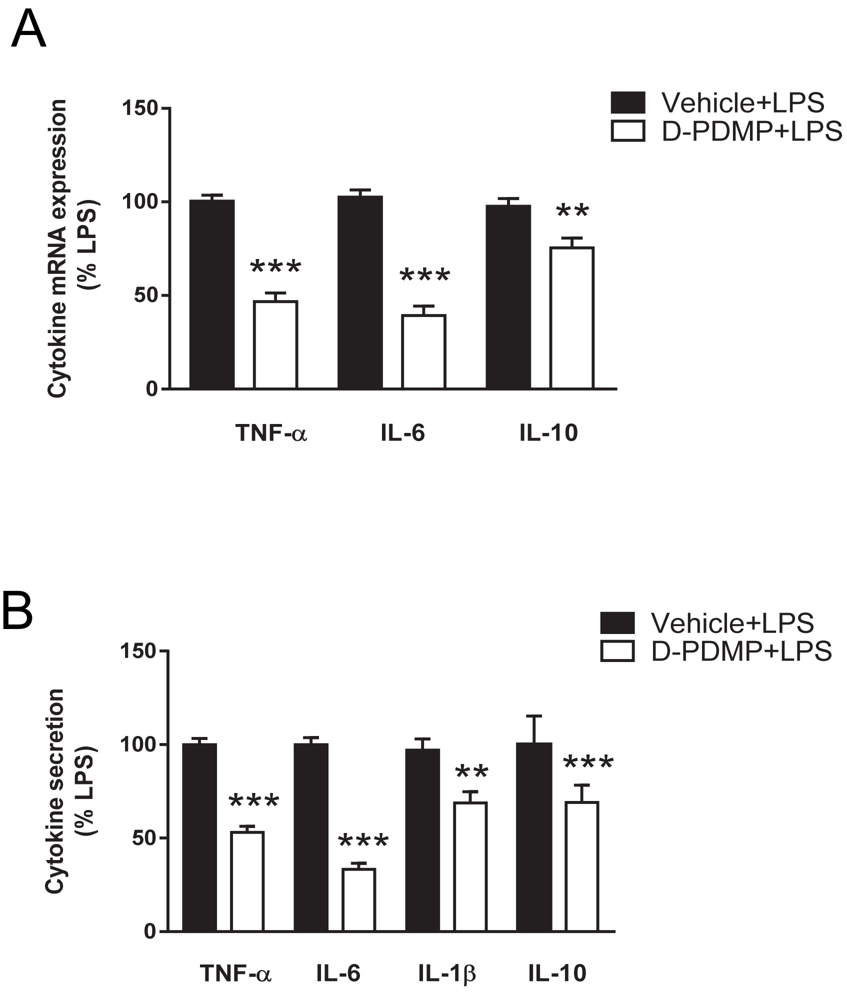

Supplementary Figure S2. **GCS inhibition results in downregulation of LPS-induced cytokine production in human blood-derived macrophages. (A)** Cytokine mRNA expression and **(B)** cytokine secretion from human blood-derived mouse macrophages stimulated with 100 ng/ml LPS from *S. typhimurium* for 18 h in the presence of GCS inhibitor (D-PDMP, 10 μM) or vehicle (ethanol). Data are mean ± SEM; **p< 0.01, *** p<0.001 versus LPS without D-PDMP, unpaired two-tailed t-test, *n* = 4.

Supplementary Figure S3

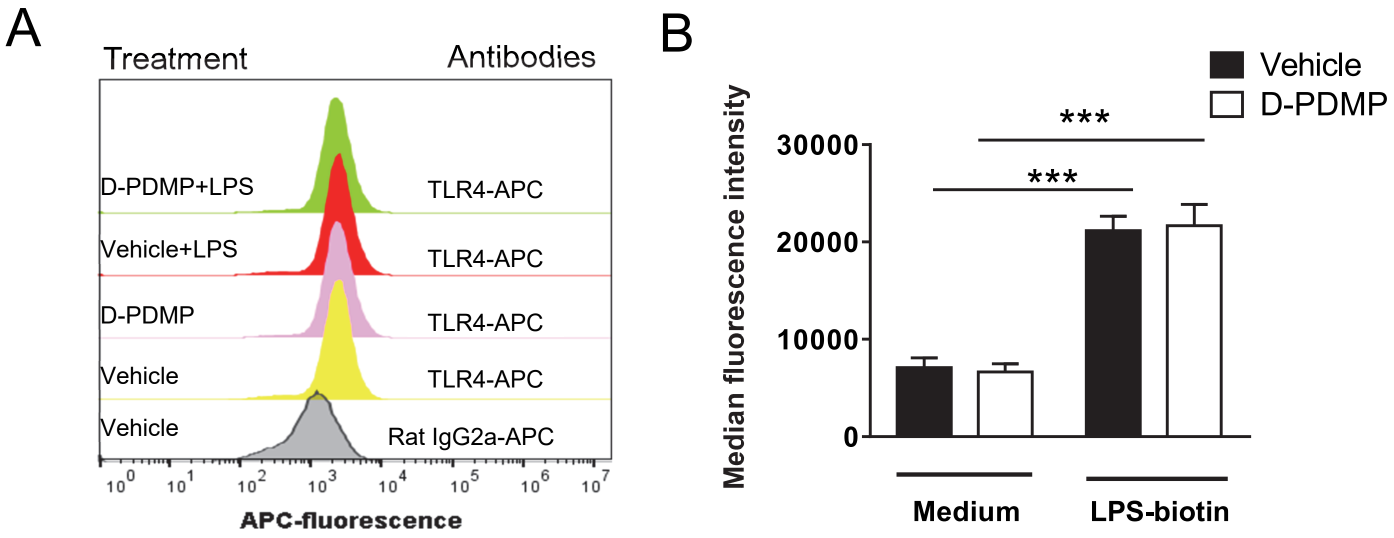

Supplementary Figure S3. GCS inhibition does not affect the TLR4 cell surface expression or LPS binding to macrophages. (A) TLR4 cell surface expression analyzed by flow cytometry in bone marrow-derived macrophages incubated for 24 h in the presence of GCS inhibitor (D-PDMP, 10 µM) or vehicle (ethanol) and thereafter stimulated with 100 ng/ml LPS from *E coli* O111:B4 for 10 min in the continued presence of D-PDMP or vehicle. Cells were stained with TLR4-APC antibodies or control isotype rat IgG2-APC antibodies as indicated in the figure. Representative histograms from one of two experiments are shown. (**B**) LPS binding to cells analyzed by flow cytometry in bone marrow-derived mouse macrophages incubated for 24 h in the presence of D-PDMP (10 µM) or vehicle and thereafter stimulated with 50 µg/ml LPS-biotin for 15 min (or cell medium as control) followed by streptavidin-Alexa fluor 488 in the continued presence of D-PDMP or vehicle. ***$p<0.001$, one-way ANOVA followed by Sidak's multiple comparisons test, $n = 3$.

Supplementary Figure S4

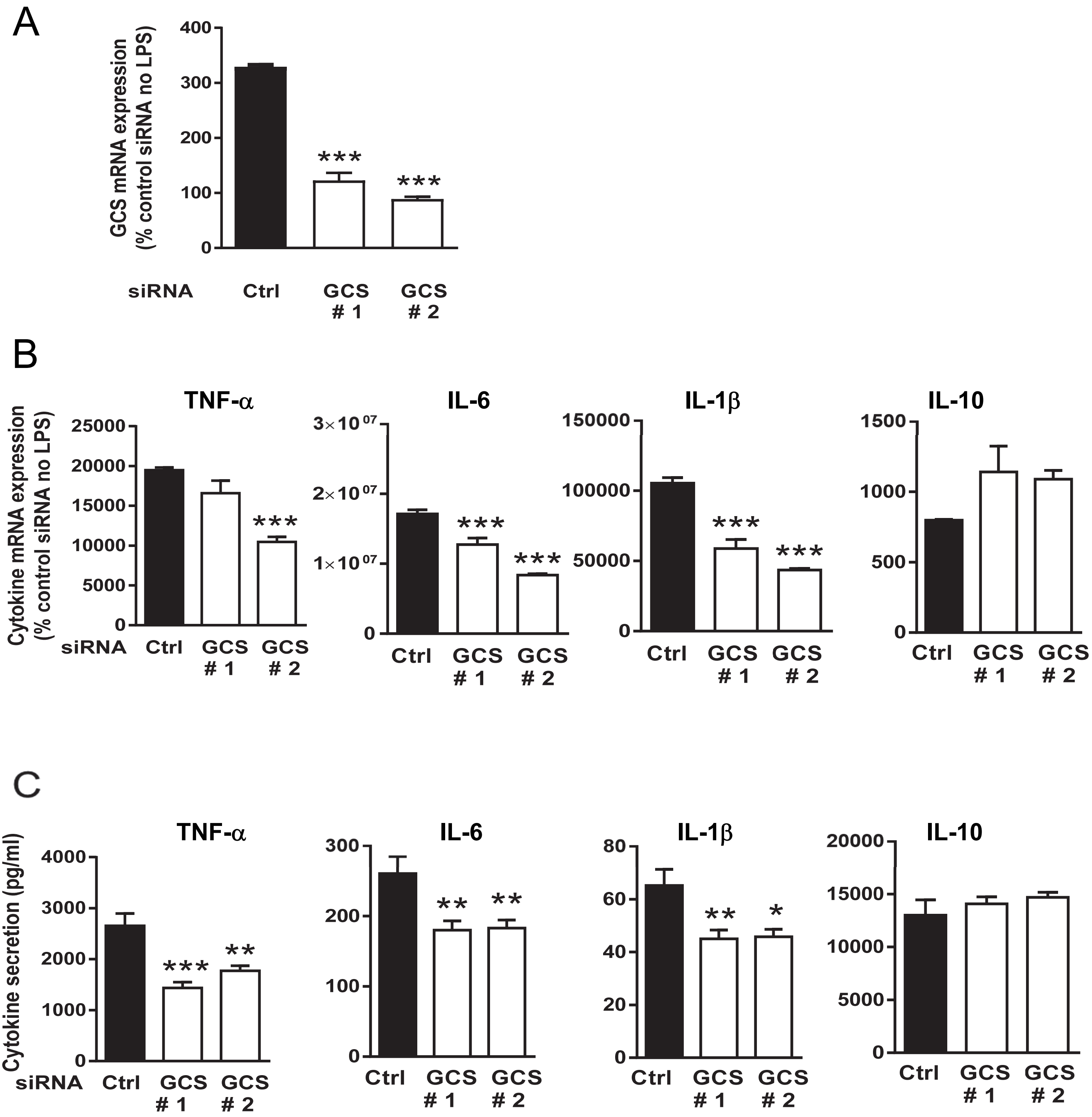

Supplementary Figure S4. GCS knockdown in bone marrow-derived mouse macrophages results in downregulation of LPS-induced cytokine production. (A) GCS mRNA expression in bone marrow-derived mouse macrophages transfected with control or GCS siRNA (#1 or #2). **(B)** Cytokine mRNA expression and **(C)** cytokine secretion from bone marrow-derived mouse macrophages transfected with control siRNA or GCS siRNA (#1 and #2) for 48 h and thereafter stimulated with 100 ng/ml LPS from *S. typhimurium* and 10 ng/ml INF-γ (or cell medium as control) for 10 h. Data are mean ± SEM; *p<0.05, **p<0.01, ***p<0.001 versus control siRNA, one-way ANOVA followed by Dunnett's multiple comparisons test (**A**) or Sidak's multiple comparisons test (**B, C**), *n* = 3.

Supplementary Figure S5

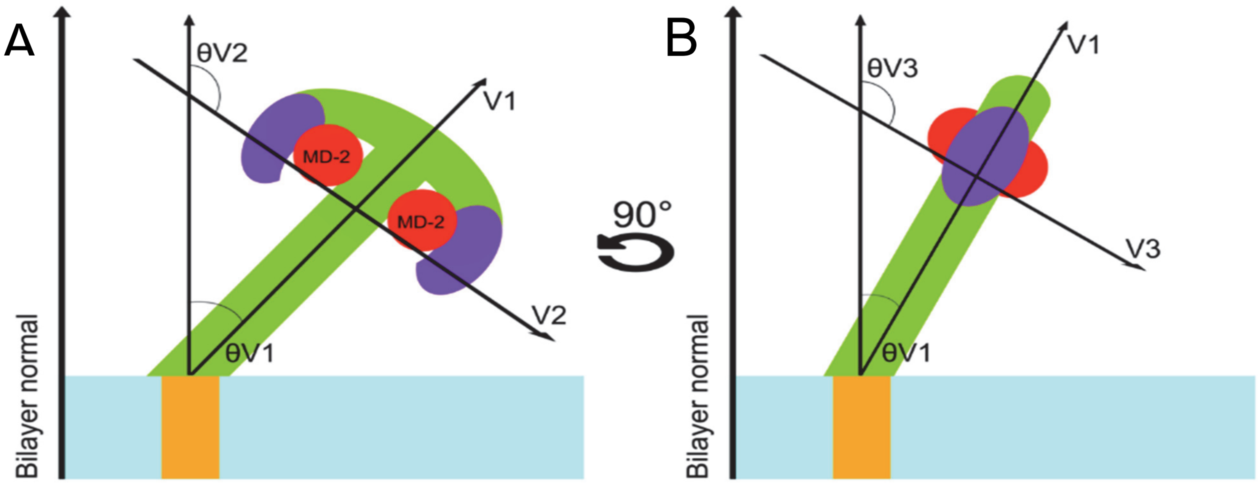

C

Mean ± EE value of the angles (degrees)				
**Nature of tilt**	**+LPS +GluCer**	**–LPS +GluCer**	**+LPS –GluCer**	**–LPS –GluCer**
**Overall tilt (ϴV1)**	32.5 ± 2.8	14.6 ± 4.8	57.0 ± 0.5	19.0 ± 0.5
**Lateral tilt (ϴV2)**	108.9 ± 1.9	93.5 ± 2.3	109.8 ± 0.7	85.4 ± 2.6
**Forward tilt (ϴV3)**	114.3 ± 2.5	77.8 ± 6.1	127.0 ± 1.1	73.2 ± 1.5

Supplementary Figure S5. Vectors used to quantify the tilts from the atomistic molecular dynamics simulations of TLR4. Schematic representation of (**A**) the 'face' and (**B**) the 'side' views of the TLR4 dimer complex showing the vectors (V1, V2 and V3) used to calculate the tilts shown in **Fig. 3**. Vector V1 connects the centers of mass of the two highest helices (residue numbers 238-250 of each monomer) and the two lowest helices (residue numbers 584-598 of each monomer) of the TLR4 complex extracellular domain 'stalk' (note that the projection of V1 onto the plane of the paper is shown). Vector V2 connects the centers of mass of the beta folds on the left and right 'lateral edges' (residue numbers 90-130 of each monomer) of the TLR4 complex extracellular domain. The 'lateral edges' of the TLR4 complex are shown in dark blue. Vector V3 is perpendicular to the plane formed by vectors V1 and V2. The angle between V1 and the membrane normal represents the 'overall' tilt between the membrane normal and the extracellular domain 'stalk'. The angle between V2 and the membrane normal represents the 'lateral' tilt. The angle between V3 and the membrane normal represents the 'forward' tilt. (**C**) The mean ± EE value of the angles (in the presence and absence of LPS in the MD-2 binding pocket and in the presence and absence of GluCer in the membrane) over the last 1 μs of simulation for 3 runs (1 run for –LPS, –GluCer).

Supplementary Figure S6

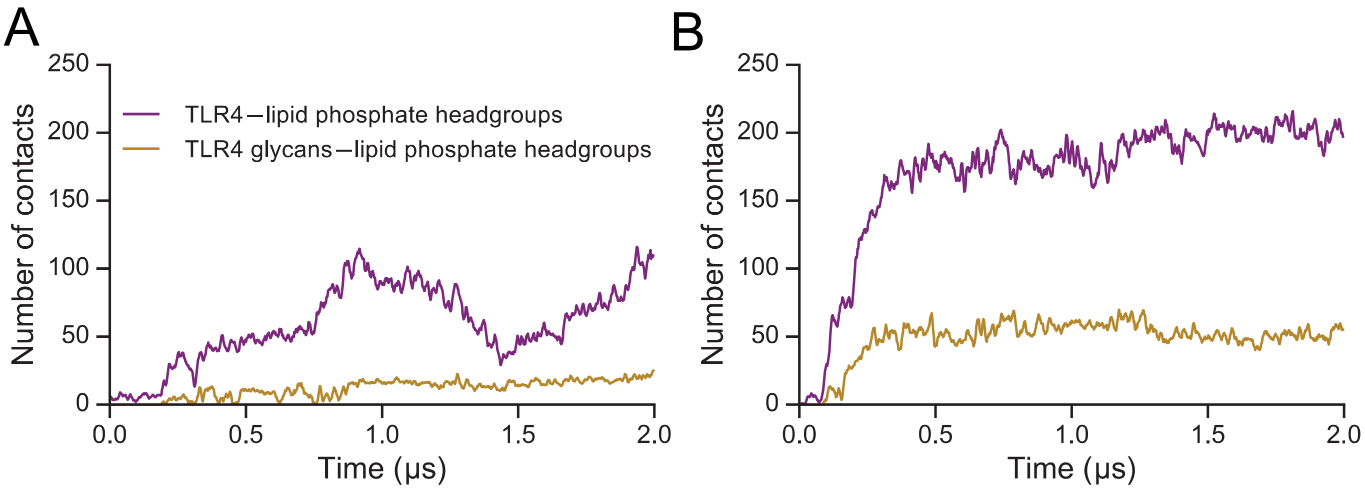

Supplementary Figure S6. Number of contacts between LPS/TLR4 and the membrane in the molecular dynamics simulations. The number of contacts (defined as pairs of atoms within a distance of 0.6 nm) between the extracellular domain residues and glycans of the LPS/TLR4 complex and the lipid phosphate headgroups of the membrane in the (**A**) presence and (**B**) absence of GluCer. Data shown are the mean of three runs. Mean ± EE values over the last 1 μs of simulation: 71.2 ± 18.3 (+LPS +GluCer) and 194.7 ± 14.1 (+LPS –GluCer) for TLR4–lipid phosphate headgroups; and 16.4 ± 2.9 (+LPS +GluCer) and 53.3 ± 7.0 (+LPS –GluCer) for TLR4 glycans–lipid phosphate headgroups. In both systems, the majority of contacts were between the TLR4 extracellular domain residues and the membrane lipid phosphate headgroups.

Supplementary Figure S7

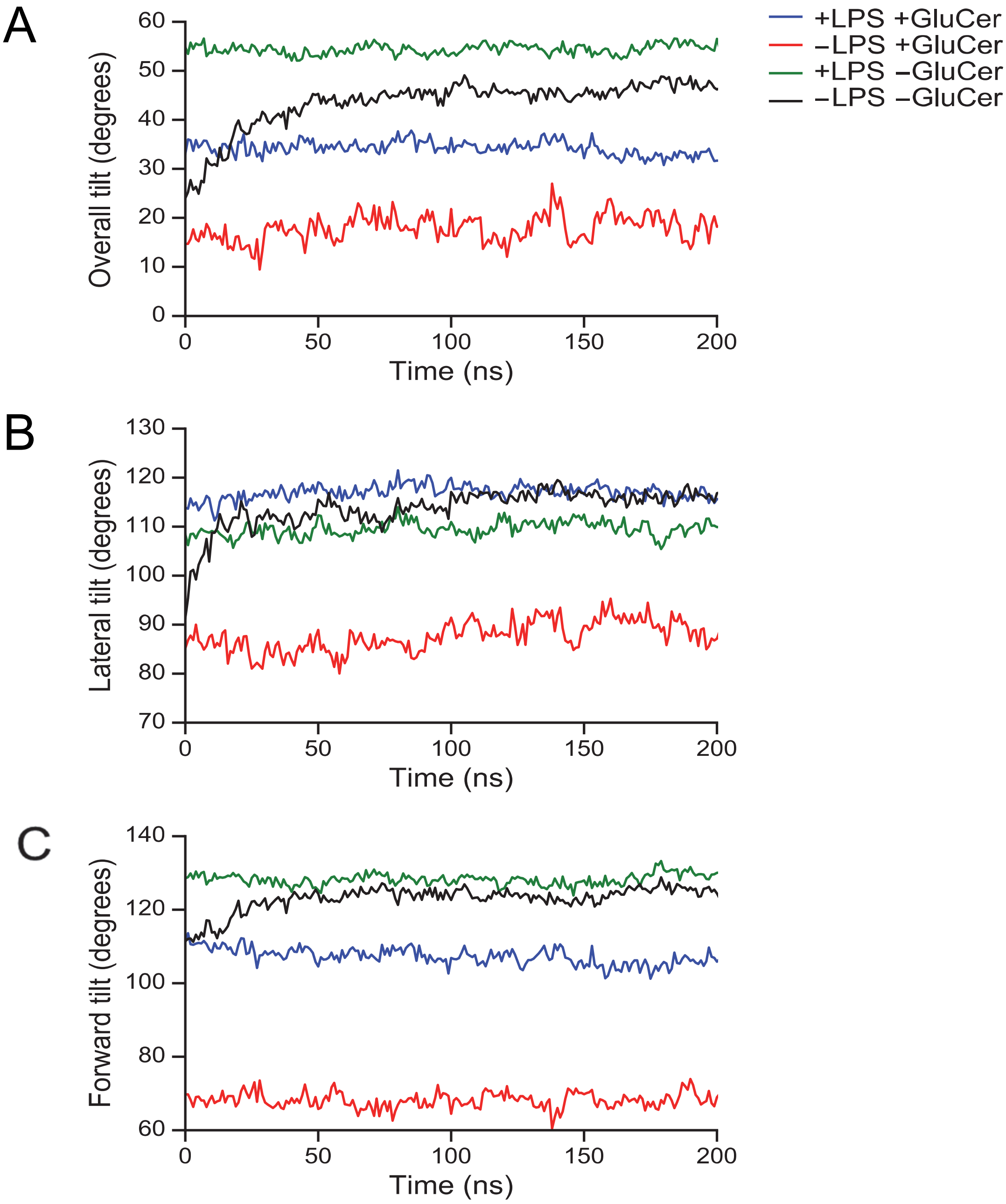

Supplementary Figure S7. Quantification of tilts from the molecular dynamics simulations of TLR4 using the reaction field method to limit the electrostatic interactions. The simulations continued for 200 ns beyond the 2 μs timepoint shown in Figure 2 and electrostatic interactions beyond a cutoff of 1.4 nm were neglected. (**A**) The 'overall' tilt, (**B**) the 'lateral' tilt, and (**C**) the 'forward' tilt of the TLR4 extracellular domain throughout the continued simulation (200 ns) for 1 run in the presence and absence of (1) LPS in the MD-2 binding pocket and (2) GluCer in the membrane.

Supplementary Figure S8

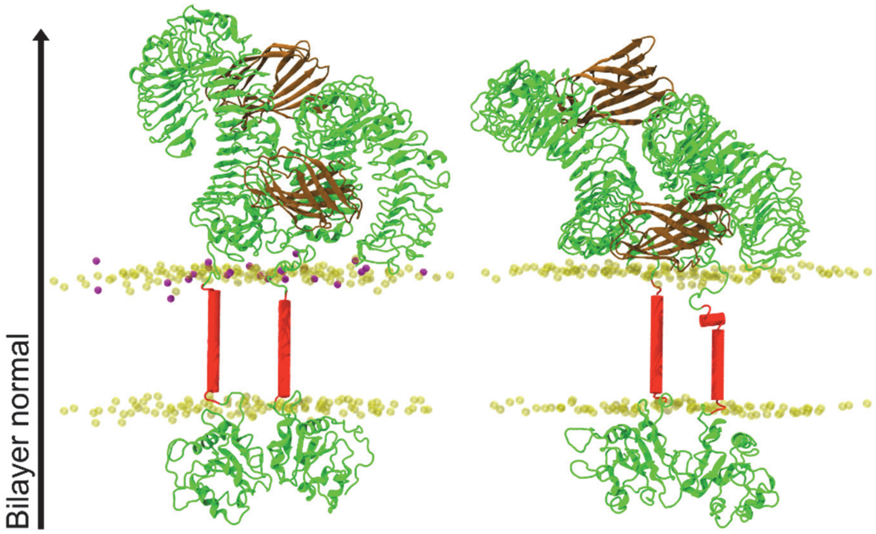

Supplementary Figure S8. Representative snapshots at 2 μs showing the conformation of the transmembrane helices of the LPS/TLR4 complex in the presence (left) and absence (right) of GluCer. MD-2 molecules are shown in brown. A deformation of one of the helices was observed between Phe647 and His651 in all 3 runs. We did not observe any obvious effect on the relative orientation of the intracellular TIR domain in our simulations, possibly because of the compact dimeric structure of the initial configuration, which remained almost unchanged throughout the whole simulation run. This part of the system is not well known from experimental sources, and assumptions had to be made while the model was constructed and assembled.

Supplementary Figure S9

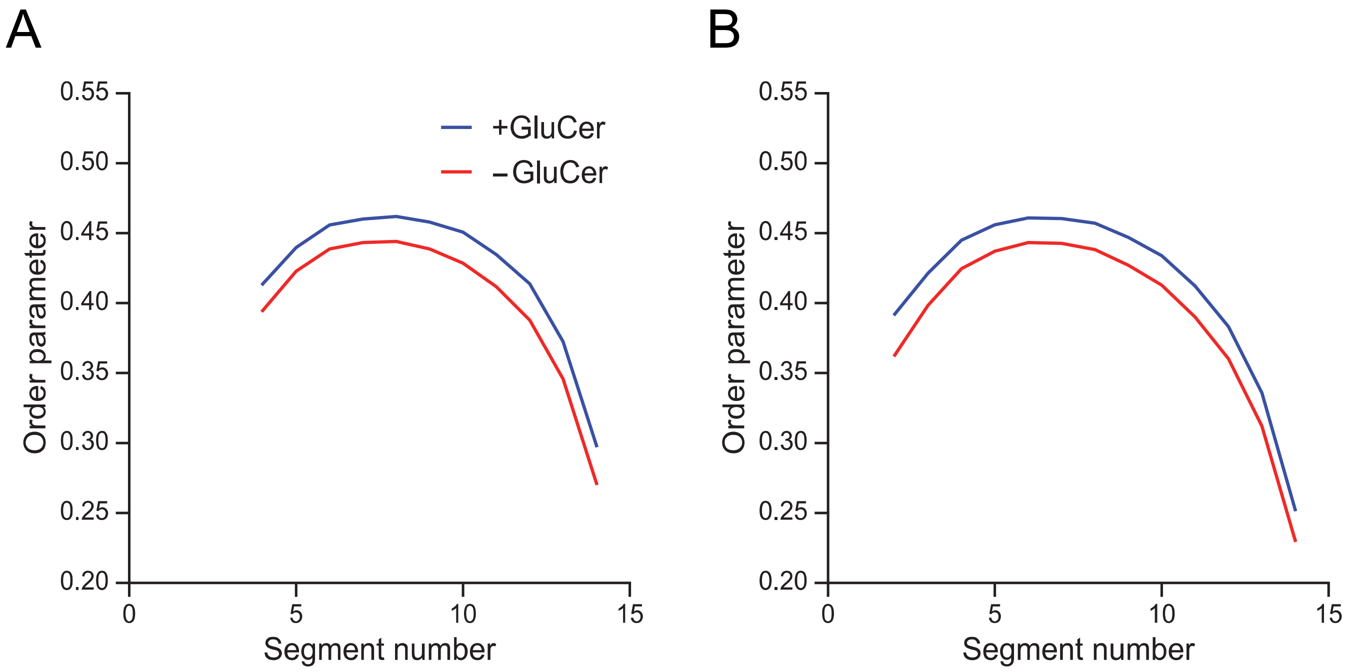

Supplementary Figure S9. Lipid order parameter. Order parameters (Scd) of (**A**) the acyl chain and (**B**) the backbone of sphingomyelin in symmetric membranes (with the LPS/TLR4 complex inserted) in the absence and presence of GluCer.

Supplementary Figure S10

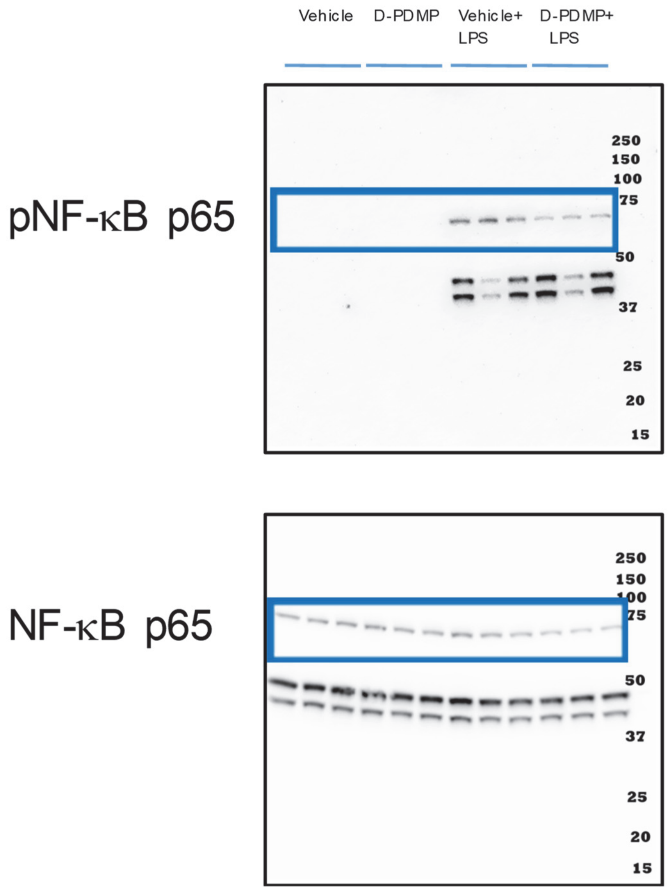

Supplementary Figure S10. Full-length blots for **Fig. 1D**. The membrane was first incubated with pNF-κB p65 antibodies and then stripped and incubated with NF-κB p65 antibodies. Images were acquired using the Fusion FX7 (Wilber Lourmat) digital camera, using optimal auto-exposure time for each antibody. The images presented in **Fig. 1D** are crops of the blot regions indicated by the blue surrounding lines. The additional bands on the membranes are a result of earlier incubation with other antibodies (pERK1/2/ERK1/2).

Supplementary Figure S11

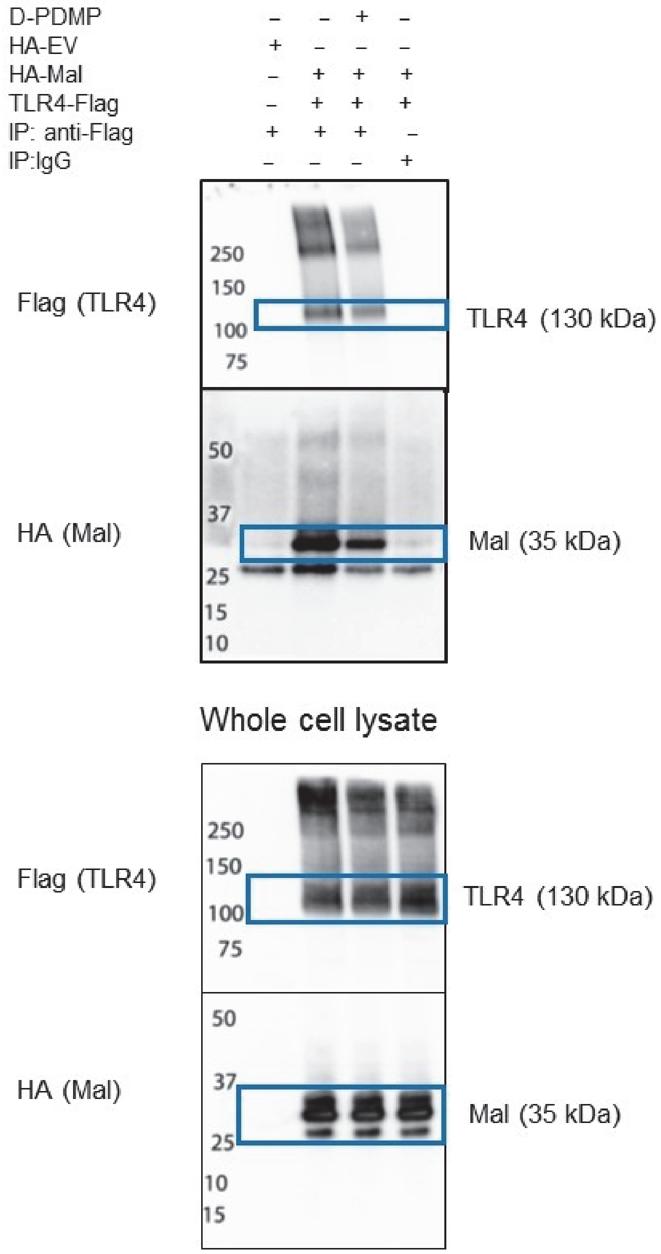

Supplementary Figure S11. Expanded blots to **Fig. 4D.** Membranes were cut between 75 and 50 kDa MW before incubation of strips with the respective primary antibodies. Images were acquired with ChemiDoc ^{TM} Touch imaging system using optimal auto-exposure times for each strip. The images presented in **Fig. 4D** are crops of the blot regions indicated by the blue surrounding lines.

Supplementary Table S1. Characteristics of the 20 models for the TIR domain created using the SWISS-MODEL modeling platform (the models to refine the final model are indicated by shading)

Model	Template	Identity	Resolution[a]	Similarity	Coverage	GMQE	QMEAN
1	1FYV.1.A	34.87	2.90 Å	0.37	0.86	0.63	-3.86
2	1FYW.1.A	38.78	3.00 Å	0.39	0.84	0.62	-5.13
3	1FYX.1.A	38.10	2.80 Å	0.39	0.84	0.62	-4.70
4	1O77.1.B	38.62	3.20 Å	0.39	0.82	0.58	-4.82
5	1O77.1.B	38.62	3.20 Å	0.39	0.82	0.61	-3.98
6	1O77.1.D	38.62	3.20 Å	0.39	0.82	0.59	-6.35
7	1O77.2.A	38.62	3.20 Å	0.39	0.82	0.58	-6.45
8	1FYW.1.A	41.13	3.00 Å	0.40	0.80	0.61	-4.75
9	1FYX.1.A	40.43	2.80 Å	0.40	0.80	0.60	-4.90
10	2J67.1.A	33.33	2.20 Å	0.36	0.84	0.59	-3.20
11	2J67.1.B	33.33	2.20 Å	0.36	0.84	0.56	-4.71
12	1FYV.1.A	41.30	2.90 Å	0.40	0.78	0.59	-5.36
13	2J67.1.A	34.75	2.20 Å	0.37	0.80	0.56	-2.80
14	2J67.1.B	34.75	2.20 Å	0.37	0.80	0.53	-4.22
15	2JS7.1.A	31.54	NMR	0.37	0.74	0.48	-7.53
16	4DOM.1.A	31.54	1.80 Å	0.37	0.74	0.49	-6.73
17	4EO7.1.A	31.54	1.45 Å	0.37	0.74	0.48	-6.25
18	2Z5V.1.A	31.54	NMR	0.37	0.74	0.49	-6.04
19	3J0A.1.A	34.13	EM	0.39	0.72	0.52	-4.01
20	3J0A.1.A	29.94	EM	0.36	0.89	0.63	-4.88

GMQE, Global Model Quality Estimation; QMEAN, statistical scoring function
[a] Resolutions for crystal structure given in ångström (Å); structures derived from nuclear magnetic resonance spectroscopy and expectation minimisation algorithm indicated by NMR and EM, respectively.

Supplementary Table S2. Glycosylated residues on TLR4

TLR4 extracellular domain	MD-2
ASN34	ASN26
ASN172	ASN114
ASN204	
ASN307	
ASN495	
ASN524	
ASN575	
ASN622	

Supplementary Table S3. Molecular composition and simulation details for each TLR4/membrane system

	Number of molecular components and simulation details			
	+LPS +GluCer	–LPS +GluCer	+LPS –GluCer	–LPS –GluCer
LPS	2	-	2	-
Phosphatidylcholine	305	305	252	252
Sphingomyelin	180	180	200	200
Cholesterol	545	545	452	452
Glucosylceramide	70	70	0	0
Phosphatidylethanolamine	130	130	112	112
Phosphatidylserine	130	130	112	112
Water	151354	151358	141420	141424
Sodium	571	567	530	526
Chlorine	409	409	386	386
Number of runs	3	3	3	1
Duration of each simulation [μs]	2	2	2	2

Supplementary Video 1. Real-time visualization (over 2 μs) of the orientational changes of the LPS/TLR4 complex in the presence of GluCer. TLR4 in green; MD-2 in red; LPS in blue; membrane lipids in grey; GluCer in purple.

Supplementary Video 2. Real-time visualization (over 2 μs) of the orientational changes of the LPS/TLR4 complex in the absence of GluCer. TLR4 in green; MD-2 in red; LPS in blue; membrane lipids in grey.
